# FiPhoPHA—A Fiber Photometry Python Package for Post Hoc Analysis

**DOI:** 10.1523/ENEURO.0221-25.2025

**Published:** 2025-08-05

**Authors:** Vasilios Drakopoulos, Alex Reichenbach, Romana Stark, Claire J. Foldi, Philip Jean-Richard-dit-Bressel, Zane B. Andrews

**Affiliations:** ^1^Monash Biomedicine Discovery Institute and Department of Physiology, Monash University, Clayton, Victoria 3800, Australia; ^2^School of Psychology, UNSW, Sydney, New South Wales 2052, Australia

**Keywords:** bootstrapping, fiber photometry, permutation tests, Python, unbiased

## Abstract

Fiber photometry is a neuroscience technique that can continuously monitor in vivo fluorescence to assess population neural activity or neuropeptide/transmitter release in freely behaving animals. Despite the widespread adoption of this technique, methods to statistically analyze data in an unbiased, objective, and easily adopted manner are lacking. Various pipelines for data analysis exist, but they are often system specific, are only for preprocessing data, and/or lack usability. Current post hoc statistical approaches involve inadvertently biased user-defined time-binned averages or area under the curve analysis. To date, no post hoc user-friendly tool with few assumptions for a standardized unbiased analysis exists, yet such a tool would improve reproducibility and statistical reliability for all users. Hence, we have developed a user-friendly post hoc statistical analysis package in Python that is easily downloaded and applied to data from any fiber photometry system. This Fiber Photometry Post Hoc Analysis (FiPhoPHA) package incorporates a variety of tools, a downsampler, bootstrapped confidence intervals (CIs) for analyzing peri-event signals between groups and compared with baseline, and permutation tests for comparing peri-event signals across comparison periods. We also include the ability to quickly and efficiently sort the data into mean time bins, if desired. This provides an open-source, user-friendly Python package for unbiased and standardized post hoc statistical analysis to improve reproducibility using data from any fiber photometry system.

## Significance Statement

Despite the widespread adoption of in vivo photometry for neuroscience research, methods to statistically analyze data in an unbiased, objective, and easily adopted manner are lacking. Various pipelines for data analysis exist, but they are often system specific, are only for preprocessing data, and/or lack usability. Current post hoc statistical approaches involve inadvertently biased user-defined time-binned averages or area under the curve analysis. Here, we have developed a standardized post hoc statistical analysis package in Python that is easily downloaded and applied to data from any fiber photometry system. This provides an open-source, user-friendly Python package for unbiased and standardized post hoc statistical analysis to improve reproducibility using data from any fiber photometry system.

## Introduction

Fiber photometry is a tool used by neuroscientists to measure population level neural activity or region- and/or cell-specific neuropeptide/transmitter release in freely moving animals (Kielbinski and Bernacka, 2024). The rapid rise in popularity of this technique is driven by the ever-expanding toolbox of genetically engineered proteins that emit fluorescent light in response to changes in intracellular calcium (a proxy for neuronal activity) or neuropeptide and neurotransmitter dynamics (Simpson et al., 2024). The most well known of these is the genetically encoded green calcium indicator, GCaMP, which is used to track neuronal activity based on changes in intracellular calcium (Lütcke et al., 2010). Typically, blue light (∼465 nm; experimental) and violet light (∼405 nm; isosbestic control) are sent into the brain through a fiber-optic cable attached to a fiber-optic implant fixed in the brain. In this way, wavelength-specific excitation of cells expressing GCaMP (or other sensors excited at 465 nm) emit light, which is then passed through a filter and detected on a photodetector. Other sensors fluoresce at other wavelengths, such as the red-shifted calcium indicator jRGECO, or the red-shifted dopamine sensor (GRAB-rDA), which have excitation wavelengths of ∼560 nm (Dana et al., 2016; Fenno et al., 2020; Zheng et al., 2024).

Before statistical comparisons can be made, several data preprocessing steps are required to artifact-correct the experimental signal. Typically, the isosbestic control signal is fitted to the experimental signal, as applied in the equation $\frac{\Delta F}{F}=\frac{\mathrm{experimental}\text{-}\mathrm{fitted}\;\mathrm{isosbestic}}{\mathrm{fitted}\;\;\mathrm{isosbestic}}$ to account for neural dynamic-independent changes in fluorescence (e.g., motion artifacts, photobleaching). Following this, a common approach is to normalize the signal via *z*-scoring, where $z=\;\frac{\left(F-F\mu \right)}{F\sigma }$, with *Fµ* and *Fσ* being the mean and standard deviation of a reference period. Various pipelines for preprocessing data from various fiber photometry systems have been made freely available for use (Akam and Walton, 2019; Bruno et al., 2021; Sherathiya et al., 2021; Conlisk et al., 2023; Murphy et al., 2023; Bridge et al., 2024).

From here, post hoc statistical comparisons can be made of peri-event signals using a variety of approaches. For peri-event analyses data is aligned to an experimental event, to assess how the data changes relative to this event. This includes user-defined summary statistics for peri-event time bins, such as area under the curve, average *z*-score, or peak *z*-score (Lerner et al., 2015; de Jong et al., 2019; Clarke et al., 2023; de Araujo Salgado et al., 2023; So et al., 2023; Reichenbach et al., 2024). However, these approaches are inadvertently biased based on user-defined time periods for analysis with potential unmet assumptions about data distribution. Thus, an easy-to-use post hoc analysis tool with fewer statistical assumptions independent from any temporal information and greater power is desperately needed to improve reliability and data reproducibility in an unbiased manner.

To address this, a previous study (Jean-Richard-dit-Bressel et al., 2020) compared alternative analysis approaches including parametric confidence intervals (CIs), nonparametric bootstrapped CIs, and permutation tests that did not require obtaining summary statistics from arbitrary time bins. These approaches permit data-driven, temporally precise identification of when peri-event signals differ from baseline or comparison signals. Furthermore, bootstrapped CIs and permutation tests are resampling methods that do not require certain assumptions about the population distribution to be met (e.g., that signals are normally distributed). A key finding of this study was that Type 1 error (false positives) could be adequately reduced without inflating Type 2 error (false negatives) by using a consecutive threshold for significance, with this threshold being critical for the validity of these techniques.

Although this study concluded bootstrapped CIs and permutation tests have highly desirable qualities for analyzing peri-event waveforms, there are no user-friendly, computationally simple, freely available packages for deploying these methods in open-source programming language like Python [existing packages (https://github.com/philjrdb/FibPhotom) have only been made available for MATLAB, a proprietary program]. Recently, another pipeline for post hoc analysis was published; however, this analysis relies on data distribution assumptions that may not accurately reflect the nonparametric, noisy, and nonlinear dynamics of a fiber photometry signal (Loewinger et al., 2025). Moreover, this approach is not user-friendly and requires data modeling, which can be computationally demanding across larger datasets. Therefore, we developed a downloadable Python package, FiPhoPHA (Fiber Photometry Post Hoc Analysis), that does not require a strong coding background to implement. FiPhoPHA serves as post hoc photometry analysis toolkit for all photometry users to improve reliability and data reproducibility in an unbiased manner. This package includes a downsampler, popular time bin analyses, bootstrapped CIs, and permutation tests.

## Materials and Methods

In the following methods and instructions section, we frequently refer to terms such as “number of resamples,” “number of permutations,” and “consecutive threshold.” We define these terms here for clarity.

Number of Resamples: The number of times random samples will be drawn with replacement to determine bootstrapped confidence intervals.

Number of Permutations: The amount of random shuffling of data to determine *p* values for permutation tests.

Consecutive Threshold: The number of individual data points in the dataset that need to be in a row to consider the data statistically significant.

The package is published and available at PyPI: https://pypi.org/project/fiphopha/. Individual files are also available on GitHub: https://github.com/VasiliDrakopoulos/fiphopha.

1. Create a virtual environment in any terminal of Python (or if you have Anaconda installed):

python -m venv (environment name) or conda create -n (environment name)

1. Install package:

pip install fiphopha

1. Run main script:

fiphopha

**Figure 1.**
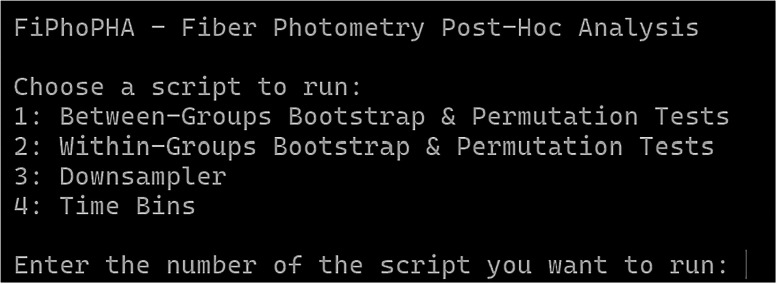
Example showing user options 1–4 for the appropriate analysis.

The text shown in Figure 1 will appear in the terminal where the user can pick 1–4 for the appropriate analysis.

### Downsampler

Downsampling reduces the amount of data used in analysis. This is particularly useful when the original sampling rate is much greater than that needed to capture neural dynamics. Different researchers downsample their signal from preprocessing by different factors, this downsampler can be coupled with other post hoc analysis to streamline processes, while still providing the temporal resolution wanted by the researcher. This downsampler works by averaging every nth data point chosen. We recommend downsampling a fiber photometry signal according to the kinetics of the sensor and computational demand. If a signal is over downsampled, there is risk of losing temporal resolution and less data points for statistical comparison. But for recordings that last several minutes to hours, this may be appropriate to offset computational demand.

### Mean time bins

Although previously described open-source fiber photometry analyses have the potential to conduct this analysis (Bridge et al., 2024), we have included this as an additional option to aid researchers in analyzing their data. This calculates the mean across specified bins, which could include a baseline period and subsequent specific time points during the comparison period after an event.

### Bootstrapped confidence intervals and permutation tests

These analysis approaches were adapted from Jean-Richard-dit-Bressel et al. (2020). They provide temporally specific alternatives to simple time bin analyses (e.g., mean time bins, area under the curve), as it reports when peri-event dynamics significantly differ from the null hypothesis. Our toolkit is set up to run bootstrapped CIs at every time point in the peri-event window to determine statistical significance. CIs are a range estimate of the true population value (e.g., the true mean fluorescence change value at event onset). Bootstrapping obtains these CIs by repeatedly resampling the data with replacement and calculating a bootstrapped estimate each time. If performed a sufficient number of times (e.g., 1,000 times), the resulting distribution of bootstrapped estimates, created from representative recombination of the dataset, serves as an approximation of the true sampling distribution, and a range for our estimate can be obtained from percentiles of this bootstrap distribution (Efron and Tibshirani, 1994). It should be noted that increasing the number of bootstraps or permutations does not narrow the CI. Rather it provides a more representative bootstrapped distribution, which asymptotes quickly; hence 2,000 bootstraps may not necessarily be better than 1,000 (Jean-Richard-dit-Bressel et al., 2020). Given this bootstrapped CI does not factor sample size, an adjustment of $\sqrt{\frac{n-1}{n}}$ has been incorporated to account for small sample narrowness bias (Hesterberg, 2015). The output provides a binary significance map; 1 denotes significance and 0 denotes no significance for a given peri-event time point, for comparisons of each group to baseline this is determined by whether the CIs include the null of 0. There are also options for between- or within-subjects analysis that includes different statistical analysis. For significance for between-subjects, the distributed means of each group are bootstrapped and subtracted. The low and high CIs from the bootstrapped distributed difference are calculated and determined if they include the null of 0. For within-subjects group comparison, each individual animal/trial is subtracted between groups and the product of the difference is bootstrapped. Due to this, for within-subjects, the same number of animals/trials in each group is needed. CIs are computed from the bootstrapped difference, and difference is determined if they include the null of 0. The choice of analyzing by animal or trial depends on the researcher's specific question. We note that unbalanced trial data can inflate the reliability of an effect but may be appropriate in certain circumstances (Editorial, 2018). Hence it is important for researchers using this package to choose between- or within-subjects comparisons using animal/trial data according to their specific data and the analysis required.

Permutation tests are another form of nonparametric resampling test that evaluates whether two groups are similar. It provides an exact *p* value for the observed difference between groups to have come from the same distribution. This compares over a distribution of data between groups for a specified period. Hence, we have incorporated in the output file of FiPhoPHA, a sheet that provides *p* values per time point between comparison periods. This nonparametric test makes few assumptions about the underlying distributions of comparison periods (Pesarin and Salmaso, 2010). These analyses have been used to compare neural dynamics signals between different events and events to a reference baseline period (Pascoli et al., 2018; Ghareh et al., 2022). It is important to note that researchers often choose different baseline periods surrounding an event, usually a timeframe before the event or the entire recording period, which will likely change the *y*-axis range considering that the neural data obtained during recording represents a relative change. This may change the results as depending on the relative change of the data, the signal may deviate differently from baseline.

### Choosing a consecutive threshold for significance

A consecutive threshold (also known as a temporal threshold) stipulates a minimum period that needs to be continuously significant for a comparison to be considered significant. This threshold is needed due to the issue of multiple comparisons and random signal crossings when analyzing all time points in the peri-event window (e.g., through bootstrap CI or permutation tests). This consecutive threshold effectively combats Type 1 errors (false positives) without substantially increasing Type 2 errors (false negatives; Jean-Richard-dit-Bressel et al., 2020). The appropriate consecutive threshold depends on several factors, including sensor kinetics and how a signal has been processed (e.g., low-pass filtering).

Sensor kinetics refer to how quickly a sensor's signal can change in response to the dynamic it tracks (i.e., rise and decay time). Rise and decay times indicate the shortest duration signal that a sensor reports and therefore represents a logical temporal lower bound for what is occurring physiologically. For example, in our demonstration of the FiPhoPHA package provided below, we used a dopamine sensor (GRAB-DA1m) that has an estimated on (rise) time of ∼0.08 s and an off (half-decay) time of ∼0.33 s (Sun et al., 2018). Therefore, real changes in dopamine concentrations are unlikely to translate to deviations in a GRAB-DA signal that occur for <0.41 s. So, 0.41 s can serve as a kinetics-based consecutive threshold that allows identification of real signal change, while limiting the detection of spurious signal change (Type 1 errors). In our example data below, we have used 400 ms for our consecutive threshold based on sensor kinetics, which is equal to four significant data points in a row when sampled at 10 Hz (referred to as consecutive threshold 4).

Low-pass filtering is used to improve signal-to-noise by removing the high-frequency signals, often associated with noise. Therefore, a consecutive threshold needs to account for any low-pass filtering applied to photometry data. Previous studies observed that a consecutive threshold of 1 s/filter Hz (the “low-pass frequency window”) was an effective rule-of-thumb for reducing family-wise Type 1 error (Jean-Richard-dit-Bressel et al., 2020). In the FiPhoPHA demonstration below, our photometry system applied a 6 Hz low-pass filter during recordings, so a consecutive threshold based on the low-pass filter should be >1/6 s (∼170 ms). To account for this in our example data below, we have used 200 ms for our low-pass filter consecutive threshold, which is equal to 2 significant data points in a row when sampled at 10 Hz (referred to as consecutive threshold 2).

A more conservative consecutive threshold could also be chosen that considers other factors such as the time taken for user-defined behavioral classification (i.e., the time taken to perform or observe and classify a change in behavior). It is important to acknowledge that this used-defined threshold is harder to objectively define when compared with a consecutive threshold based on low-pass filtering or sensor kinetics and may not always be appropriate. In our example data below, we have user-defined consecutive threshold of 500 ms, which is equal to 5 significant data points in a row when sampled at 10 Hz (referred to as consecutive threshold 5). Our user-defined threshold of 500 ms is based on the time required for a mouse to collect and consume a 20 mg sucrose pellet.

Given multiple factors can be used to determine a consecutive threshold, we recommend choosing the more conservative (longer duration) threshold between the kinetics of the sensor and the low-pass filter frequency. For the examples described above, the temporal threshold based on kinetics (400 ms: consecutive threshold 4 when data are sampled at 10 Hz) should be chosen over the low-pass filter threshold (200 ms: consecutive threshold 2 when data are sampled at 10 Hz). To show how temporal thresholds influence results, we have presented data at 95% and 99% CI with and without the consecutive thresholds described above (Figs. 23, 24).

**Figure 2.**
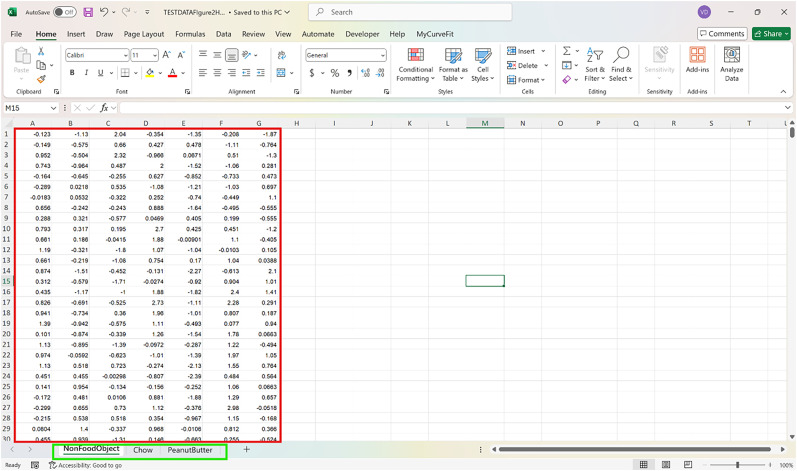
Example showing how the dataset should be represented in Excel.

**Figure 3.**
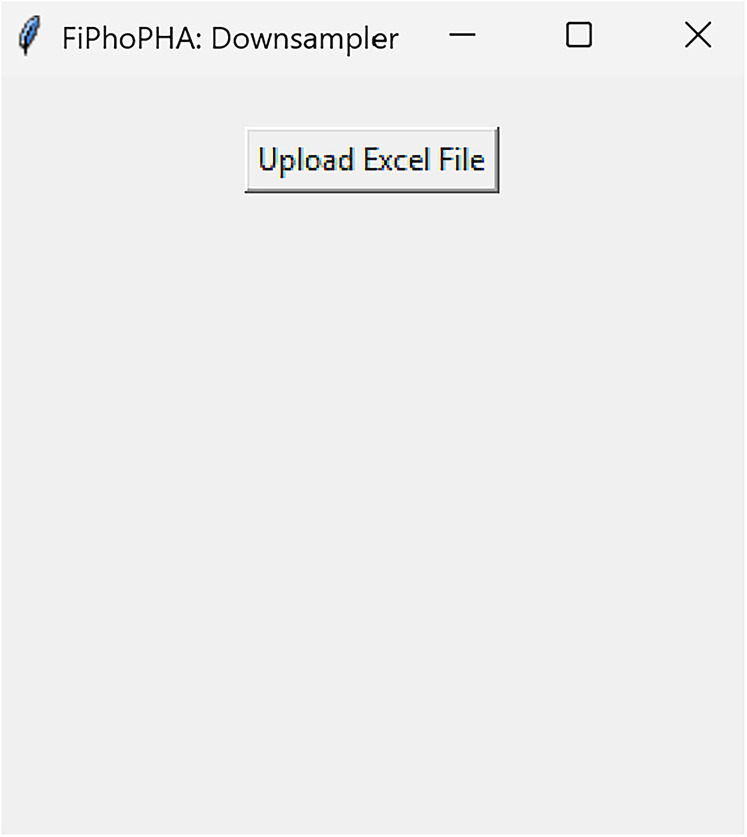
Example showing how the graphical user interface (GUI) will be presented.

Importantly, the data sampling frequency should be considered when applying a consecutive threshold to downsampled data. For example, applying a consecutive threshold of 10 to data sampled at 10 Hz (i.e., 1 data point every 100 ms, consecutive threshold is 10 × 100 ms) would be the same as a consecutive threshold of 1 when downsampled to 1 Hz (i.e., 1 data point every 1,000 ms, 1 × 1,000 ms).

### Instructions

The following protocol is to install the FiPhoPHA Python package and provides instructions on the setup by using example data from Reichenbach and colleagues, randomly selected data that uses three groups of data (Fig. 2*H*; Reichenbach et al., 2022). The example uses the within-subjects analysis as it uses the same animals for three trials/events of dopamine release in the nucleus accumbens from a nonfood object, chow, and peanut butter. A Windows, Mac, or Linux computer operating system is required with Python installed https://www.python.org/downloads/ and Microsoft Excel https://www.microsoft.com/en-au/microsoft-365/excel.

For all analyses arrange data in Excel as follows:

Each sheet in the Excel document represents a group of data and each column is a different animal/trial as seen in the red and green in Figure 2. The time points of the dataset are now represented by the row index of the Excel document.

**Figure 4.**
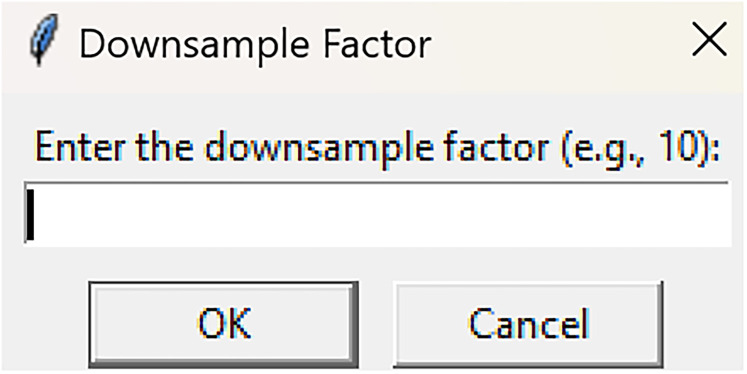
Example showing how GUI will be presented for the downsample factor to be selected.

1. Downsampler
  1. Run fiphopha script and select 3: Downsampler.
  2. The graphical user interface (GUI) will be presented in Figure 3.
  3. Press upload Excel file and select your Excel file.
  4. The GUI will be presented in Figure 4 for the downsample factor to be selected.

Since the following example data is sampled every 99.8 ms (∼10 Hz), we will provide an example of downsampling by a factor of 10, to get an output of ∼1 Hz.

**Figure 5.**
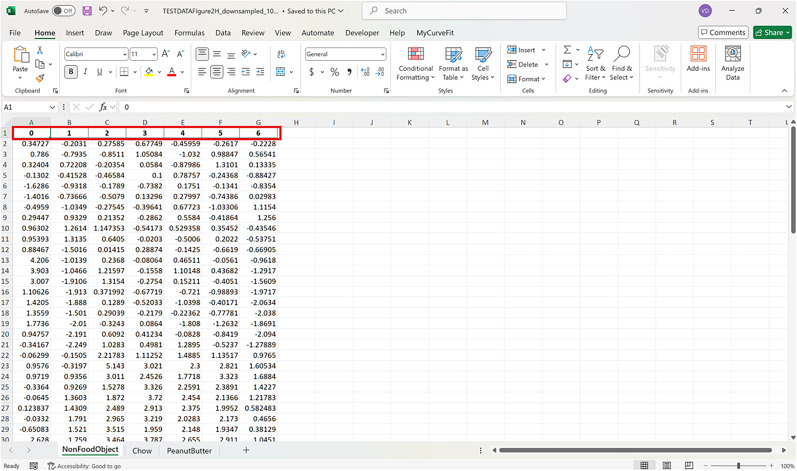
The red box shows the indexed columns of the output file to specify how many animals/trials the code analyzed per sheet.

1. The output file, shown in Figure 5, saved as the original Excel document name_downsampled_10 will show in the same directory as the input file.

The red box shows the indexed columns of the output file to specify how many animals/trials the code analyzed per sheet.

**Figure 6.**
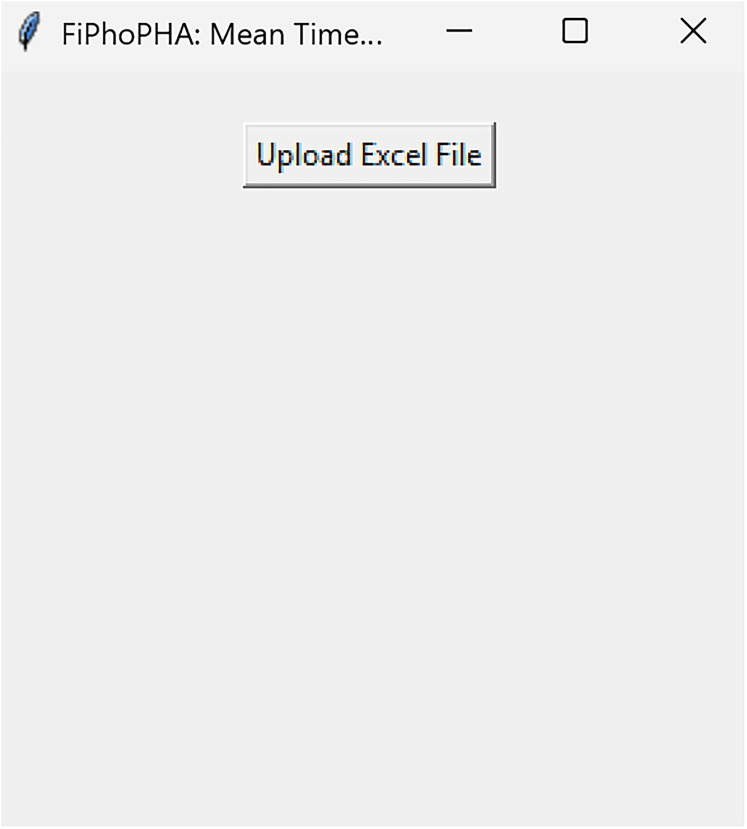
When running Mean Time Bins the following GUI will be presented.

**Figure 7.**
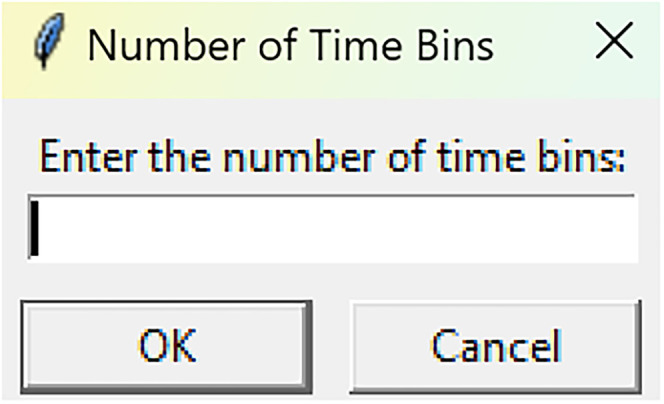
The following GUI will be presented for the number of time bins.

**Figure 8.**
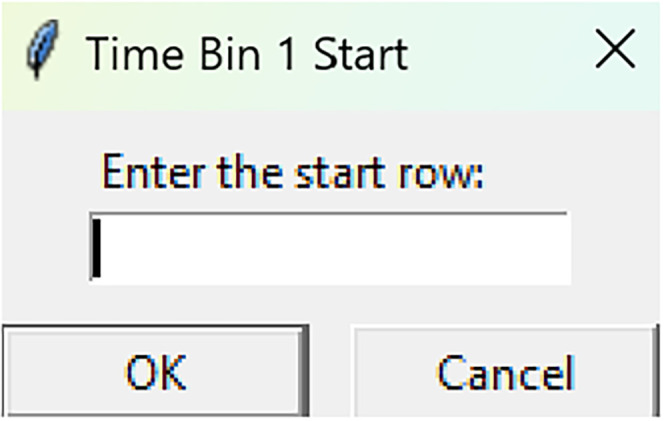
After number of time bins are selected, the following GUI is presented to enter the start row of the 1st time bin.

**Figure 9.**
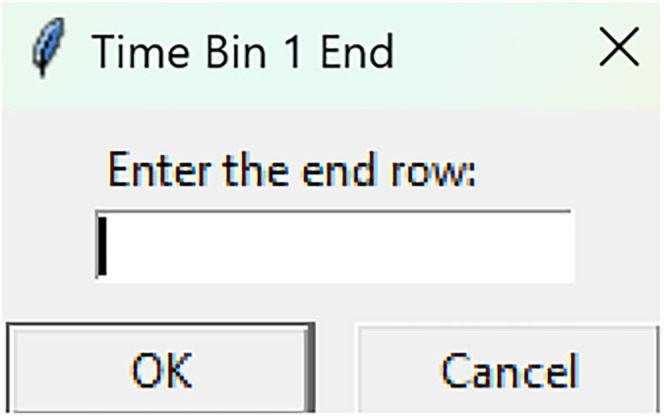
The following GUI is shown to enter the end row of the 1st time bin.

**Figure 10.**
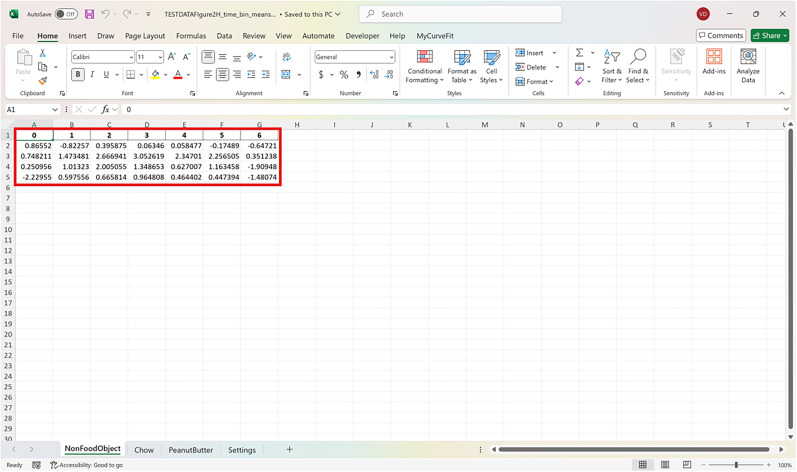
The red box shows the indexed columns of the output file to specify how many animals/trials the code analyzed per sheet with the mean time bins underneath.

1. Mean time bins
  1. Run fiphopha script and select 4: Time bins.
  2. The GUI will be presented in Figure 6.
  3. Press upload Excel file and select your Excel file.
  4. The GUI shown in Figure 7 will be presented for the number of time bins. Since the example data used presents 813 time points between −20 s before event and 60 s after event, we have selected four time bins of ∼20 s each. In this example, our data is sampled at 10 Hz so the time bins for 20 s intervals are 0–212, 213–416, 417–619, and 620–812 for the Excel row index. The max value is 1 less than the max value due to Python conventions of 0 being Excel row index 1.
  5. After number of time bins are selected, the GUI shown in Figure 8 is presented to enter the start row of the 1st time bin.
  6. After, the GUI in Figure 9 is shown to enter the end row of the 1st time bin.
  7. Repeat steps 5–6 for each time bin until all are entered.
  8. After all are entered, an output Excel file will be saved to the same directory as the input file with the title of the input file with name_time_bin_means (Fig. 10).

**Figure 11.**
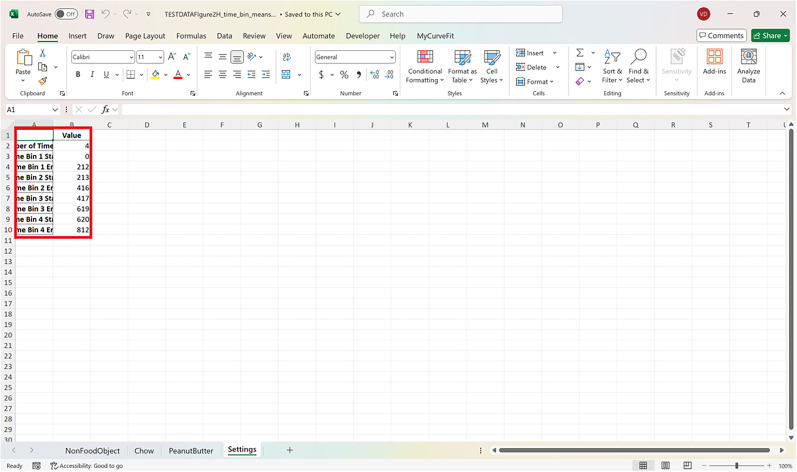
An example sowing how the settings of the number of time bins and the Excel row index of each time bin is outputted in the settings sheet of the Excel file.

The red box in Figure 11 shows the indexed columns of the output file to specify how many animals/trials the code analyzed per sheet with the mean time bins underneath.

The settings of the number of time bins and the Excel row index of each time bin is outputted in the settings sheet of the Excel file.

**Figure 12.**
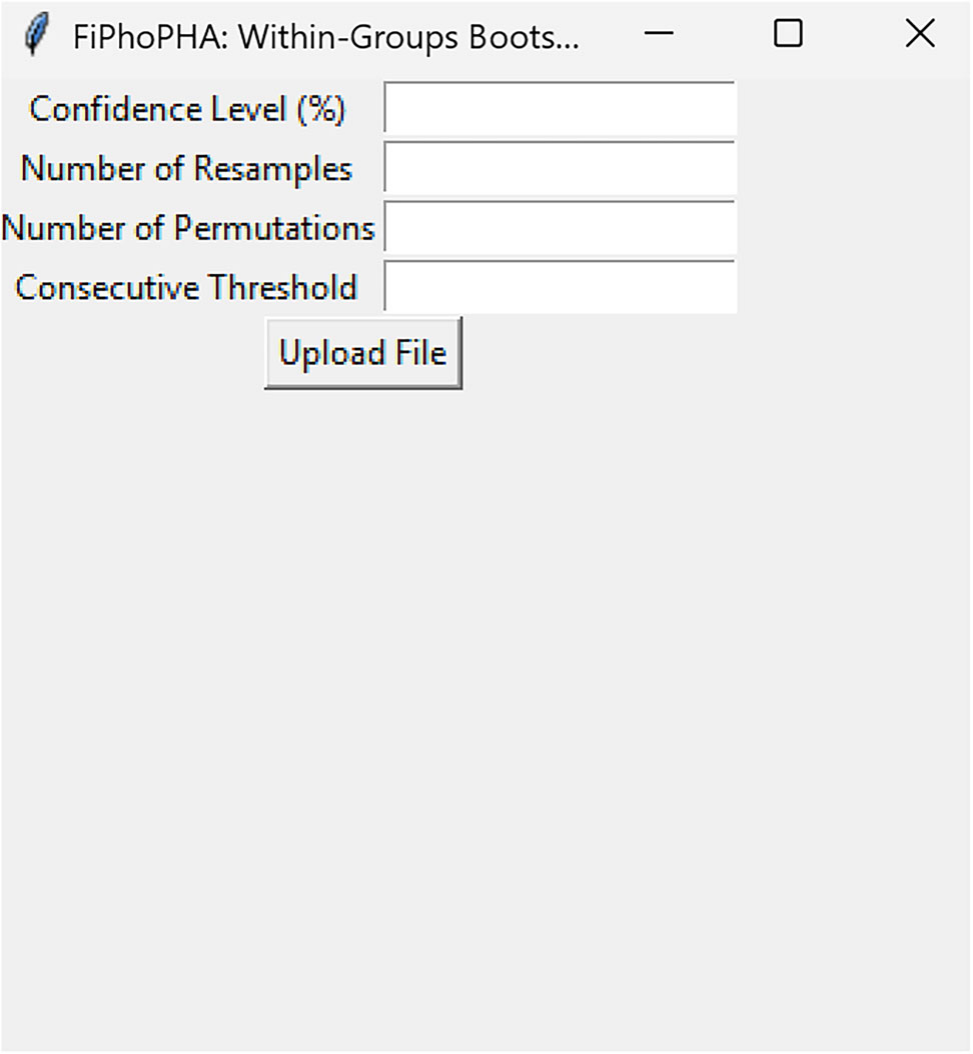
When running bootstrapped confidence intervals and permutations tests the following GUI will be presented.

**Figure 13.**
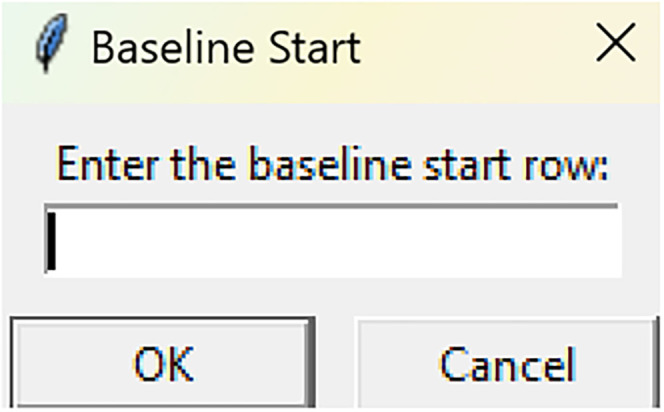
The following GUI will be shown to select the start row of the baseline.

**Figure 14.**
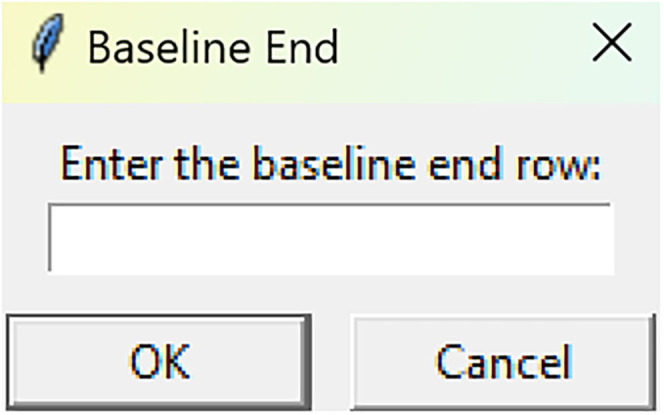
The following GUI indicates how the baseline end is selected.

**Figure 15.**
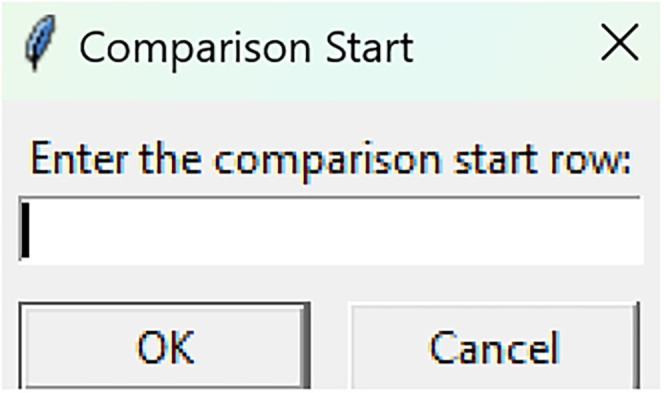
The following GUI shows the start of the comparison period.

**Figure 16.**
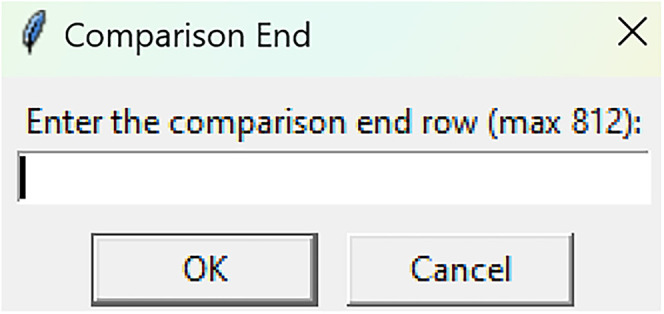
The following GUI shows the end of the comparison period.

**Figure 17.**
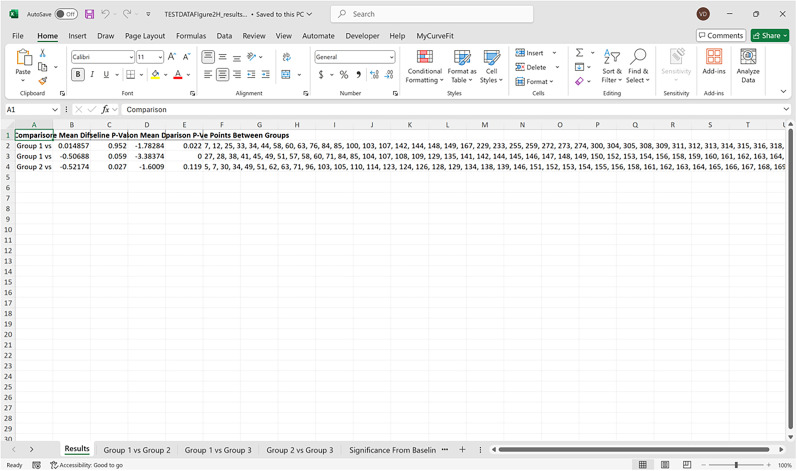
An example of the output Excel file, which will be saved to the same directory as the input file with the title of the input file with name_results.

**Figure 18.**
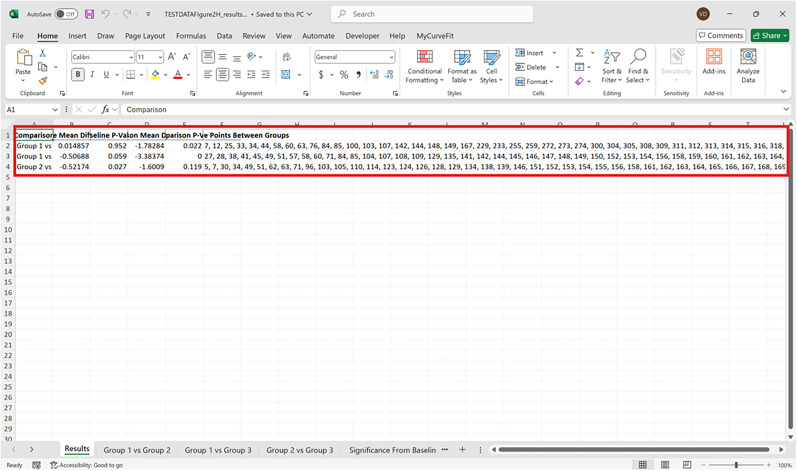
An example of the output file showing the comparison between each group of the input sheet, the mean difference between the groups and *p* values of the permutation tests of the defined baseline window, and the same with the comparison period. The last column presents all significant time points between the groups.

**Figure 19.**
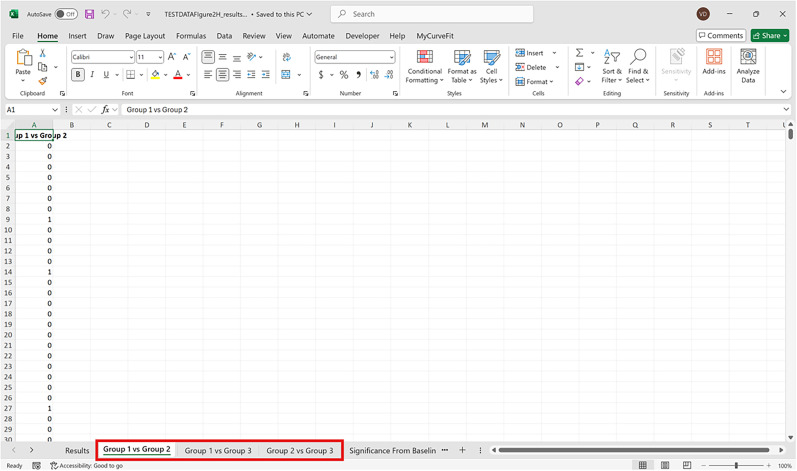
An example of a binary significance map of either 0 or 1, with 1 denoting significance for each time point across the group comparisons.

**Figure 20.**
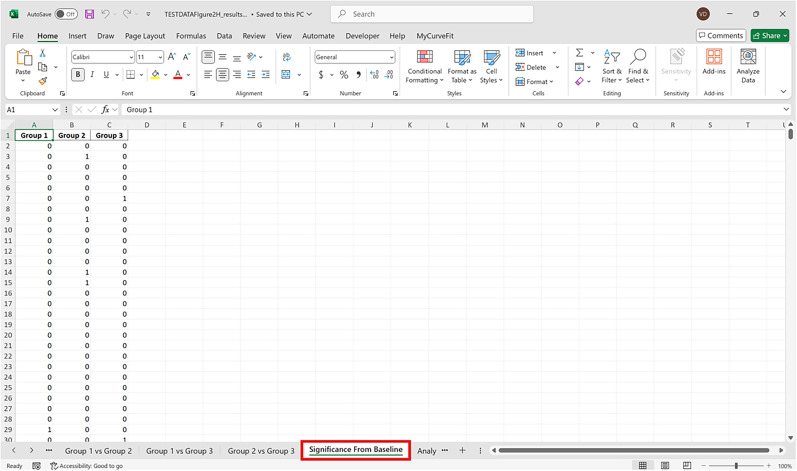
An example of a sheet displaying the significance from baseline for the groups separately, where 1 denotes significance as shown below.

**Figure 21.**
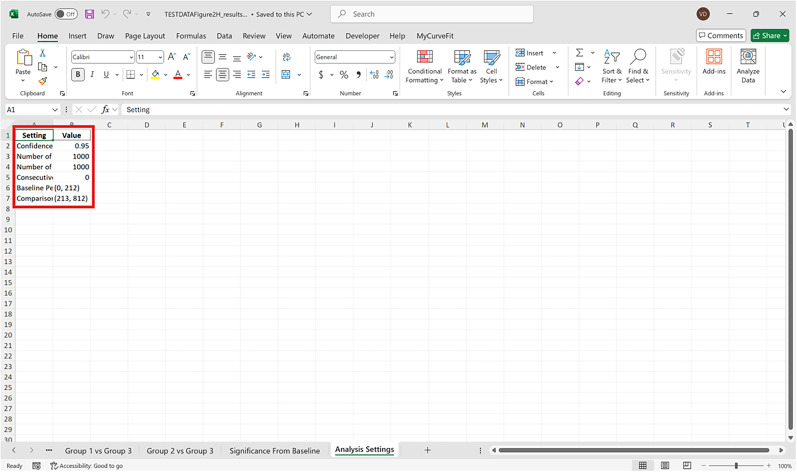
An example showing the analysis settings that the user defined in the initial GUI.

1. Bootstrapped confidence intervals and permutation tests
  1. Run fiphopha script and select appropriate analysis, in this case it is 2: Within-Groups Bootstrap & Permutation Tests.
  2. The GUI will be presented in Figure 12.
  3. Select confidence level, number of resamples, number of permutations, and consecutive threshold, then click upload file and select your input file. We used confidence levels 95% or 99%, and a standard number of 1,000 resamples, 1,000 permutations based on the previously validated measures of statistical accuracy (Jean-Richard-dit-Bressel et al., 2020). For a consecutive threshold, we used 2 (low-pass filter) or 4 (kinetics) or 5 (standardized), where our data were sampled at ∼10 Hz (i.e., every 100 ms); therefore, the consecutive threshold represents 2, 4, and 5 sampling periods, respectively (i.e., 200, 400, and 500 ms).
  4. The GUI in Figure 13 will be shown to select the start row of the baseline, 0 was selected.
  5. Then baseline end is selected, 212 was used (Fig. 14).
  6. After a GUI is shown to enter the start of the comparison period, 213 was used (Fig. 15).
  7. A GUI is then shown to enter the end of the comparison period, a max value is shown. This is the maximum row of the data minus 1, as 0 is denoted as row 1 of the Excel spreadsheet. 812 was used (Fig. 16).
  8. An output Excel file will be saved to the same directory as the input file with the title of the input file with name_results as shown below (Fig. 17).
  9. This shows the comparison between each group of the input sheet, the mean difference between the groups and *p* values of the permutation tests of the defined baseline window, and the same with the comparison period. The last column presents all significant time points between the groups (Fig. 18).
  10. The sheet in Figure 19 shows a binary significance map of either 0 or 1, with 1 denoting significance for each time point across the group comparisons.
  11. The sheet in Figure 20 displays the significance from baseline for the groups separately, where 1 denotes significance as shown below.
  12. The last sheet shown in Fig. 21 shows the analysis settings that the user defined in the initial GUI.

### Common errors

Non-numeric data found in Excel sheets or input file is empty.

When picking the row index for analysis, numbers must be non-negative and the data to be analyzed should be equal to or less than the maximum number of rows (refer to box 14–16).

For within-groups analysis, ensure multiple groups have the same number of animals/trials in each Excel sheet as each of them are subtracted point by point. If the experiment has been conducted within-groups but due to reasons there are different amounts of animals/trials in each group, it is appropriate to run the between-groups analysis, as it subtracts the bootstrapped distributions of each group.

Input/output directory is linked to a cloud file. The input/output directory must be from hard drive storage on the computer.

## Results

**Figure 22.**
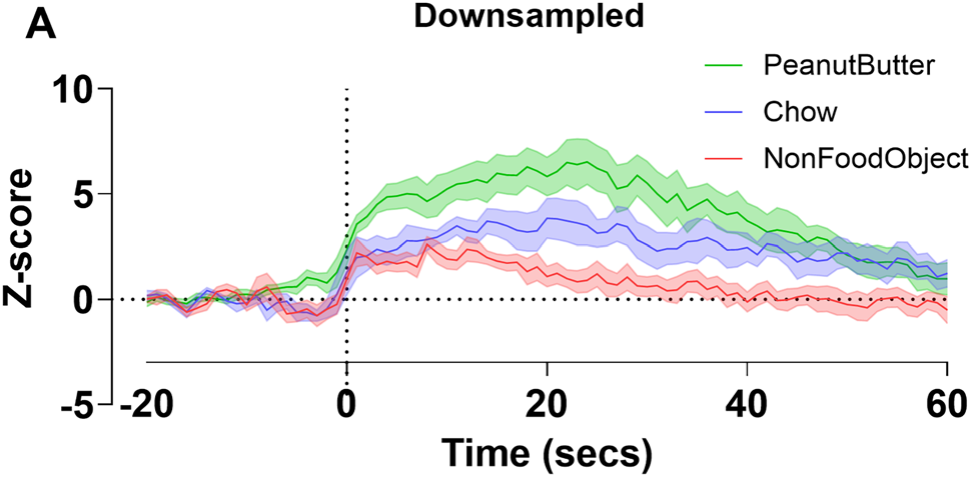
Wild-type overnight fasted nucleus accumbens dopamine release downsampled. Downsampled (by a factor of 10) *z*-score photometry trace to ∼1 Hz for nonfood object, chow, and peanut butter put in cage at time 0 (***A***). Data is represented as mean ± standard error of the mean (SEM) and *n* = 7 in all groups.

To show the utility of our approach, we have re-examined data from Reichenbach et al. (2022), with data representing dopamine release in the nucleus accumbens in overnight fasted wild-type mice with time 0 representing the placement of an object or food into the cage. The data were downsampled to 1 Hz, whereas the original data processed at 10 Hz (Fig. 22*A*).

### Bootstrapping CIs

#### Differences from baseline

**Figure 23.**
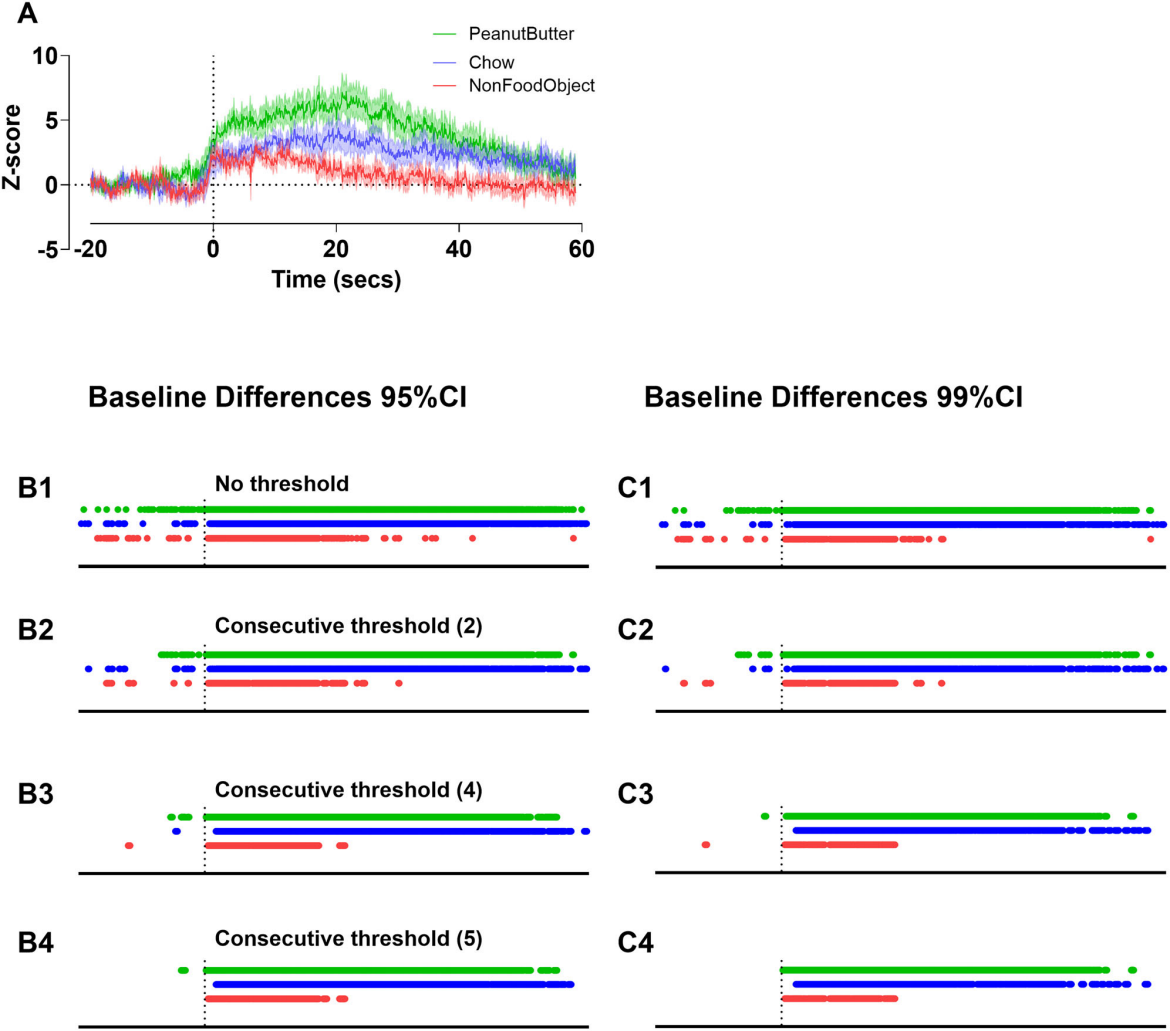
Baseline comparisons in nucleus accumbens dopamine release using bootstrapped CIs with different consecutive thresholds. Original *z*-score photometry trace for nonfood object, chow, and peanut butter put in cage at time 0 (***A***). Baseline differences from bootstrapped CIs with 1,000 resamples at 95% confidence with no consecutive threshold (***B1***). Baseline differences from bootstrapped CIs with 1,000 resamples at 95% confidence at consecutive threshold 2 (2 significant data points in a row; ***B2***). Baseline differences from bootstrapped CIs with 1,000 resamples at 95% confidence at consecutive threshold 4 (4 significant data points in a row; ***B3***). Baseline differences from bootstrapped CIs with 1,000 resamples at 95% confidence at consecutive threshold 5 (5 significant data points in a row; ***B4***). Baseline differences from bootstrapped CIs with 1,000 resamples at 99% confidence with no consecutive threshold (***C1***). Baseline differences from bootstrapped CIs with 1,000 resamples at 99% confidence at consecutive threshold 2 (2 significant data points in a row; ***C2***). Baseline differences from bootstrapped CIs with 1,000 resamples at 99% confidence at consecutive threshold 4 (4 significant data points in a row; ***C3***). Baseline differences from bootstrapped CIs with 1,000 resamples at 99% confidence at consecutive threshold 5 (5 significant data points in a row; ***C4***). Data is represented as mean ± SEM and *n* = 7 in all groups (***A***).

Using the bootstrapped CIs code, we analyzed changes in nucleus accumbens dopamine release from baseline after contact with a nonfood object, chow, and peanut butter at the original preprocessing of 10 Hz (Fig. 22*A*). With 95% CI, individual time points indicate a significance deviation from 0 (baseline) for each group where no consecutive threshold has been applied (Fig. 23*B1*). As the consecutive threshold increases (the amount of time points that need to be significant in a row), the amount of time points that are deemed significant decreases. At the 95% CI as the threshold increases, no time points pre-event are significant at a consecutive threshold of five time points except for in the peanut butter group (Fig. 23*B4*). The general trends are conserved between the 95% and 99% CI, but at every consecutive threshold there are fewer significant points at 99% CI. At the standardized five consecutive points for the 99% CI, there are no points in the pre-event baseline period that are significant (Fig. 23*C4*).

#### Differences between groups

**Figure 24.**
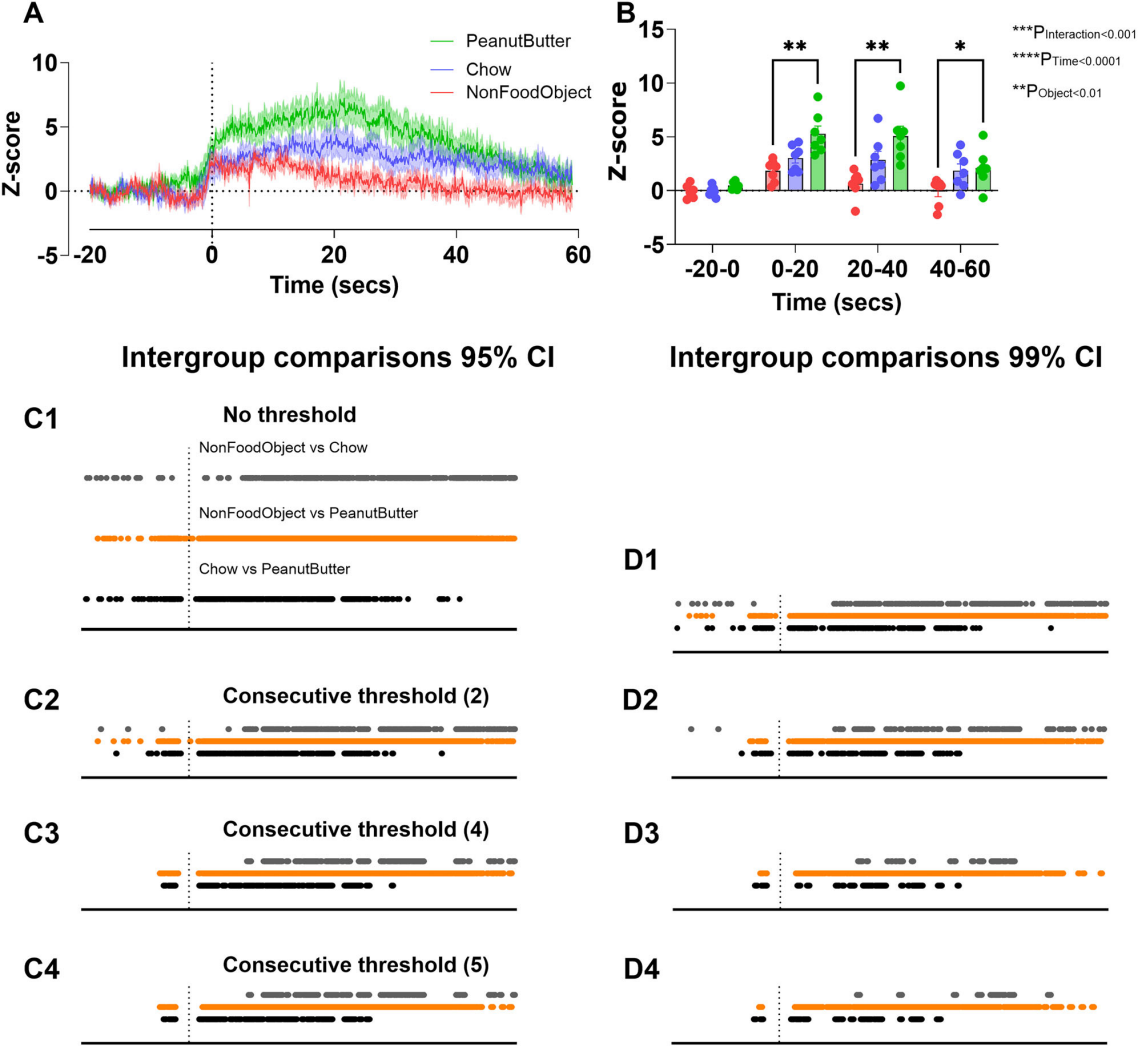
Intergroup differences in nucleus accumbens dopamine release at different consecutive thresholds using bootstrapped CIs. Original *z*-score photometry trace for nonfood object, chow, and peanut butter put in cage at time 0 (***A***). Mean time bins of 20 s each of photometry trace with main effects of interaction, time, and object using RM two-way ANOVA (***B***). Intergroup differences (nonfood object vs chow, nonfood object vs peanut butter, and chow vs peanut butter) from bootstrapped CIs with 1,000 resamples at 95% confidence with no consecutive threshold (***C1***). Intergroup differences from bootstrapped CIs with 1,000 resamples at 95% confidence at consecutive threshold 2 (***C2***). Intergroup differences from bootstrapped CIs with 1,000 resamples at 95% confidence at consecutive threshold 4 (***C3***). Intergroup differences from bootstrapped CIs with 1,000 resamples at 95% confidence at consecutive threshold 5 (***C4***). Intergroup differences from bootstrapped CIs with 1,000 resamples at 99% confidence with no consecutive threshold (***D1***). Intergroup differences from bootstrapped CIs with 1,000 resamples at 99% confidence at consecutive threshold 2 (***D2***). Intergroup differences from bootstrapped CIs with 1,000 resamples at 99% confidence at consecutive threshold 4 (***D3***). Intergroup differences from bootstrapped CIs with 1,000 resamples at 99% confidence at consecutive threshold 5 (***D4***). Two-way repeated-measures ANOVA with Tukey's multiple comparisons (***B***). Data is represented as mean ± SEM and *n* = 7 in all groups (***A***, ***B***), *p < 0.05, **p < 0.01.

We also analyzed this data using the within-subjects bootstrapped CIs code to assess intergroup comparisons in nucleus accumbens dopamine release (Fig. 24*A*). However, as a point of comparison, we first averaged data into 20 s time bins using the mean time bins code (Fig. 24*B*) and analyzed using two-way repeated-measures ANOVA with Tukey's post hoc. This approach is the current standard in the field and gave significant differences between the nonfood object and peanut butter during each of the 20 s time intervals after the event and main effects for object (*p* < 0.01), time (*p* < 0.0001), and an interaction (*p* < 0.001). With 95% CIs with no consecutive threshold (0), all groups showed significant differences during the baseline and post-event periods. With no consecutive threshold, the largest intergroup comparisons were in the nonfood object versus peanut butter group comparisons (Fig. 24*C1*). This is followed by the nonfood object versus chow comparison and then by the chow versus peanut butter comparison. When compared with the results from average time bins (Fig. 24*B*), there is clearly the need for an objective consecutive threshold. The same is shown in the 95% CI at a consecutive threshold of 2, but with fewer time points of significance (Fig. 24*C2*). As the consecutive threshold increases to 5, there are fewer points of significance with the same relative trends (Fig. 24*C4*). At the 99% CI, there are overall fewer time points of significance for all intergroup comparisons. For this confidence, at a standardized consecutive threshold of five time points, there remains significant differences in all three intergroup comparisons with the nonfood object versus peanut butter comparison having the most differences. The other two intergroup comparisons have fewer points of significance that remain less consecutive compared with using lower thresholds (Fig. 24*D4*).

### Permutation tests

The permutation tests compare the distributions of the data in either the entire baseline (pre-event) or comparison (post-event) period. Hence changing the consecutive threshold should have little to no effect on these tests. The *p* values obtained from the permutation tests represent the probability that the two samples share the same distribution. During the baseline period, *p* values change slightly as the consecutive threshold increases (Table 1) at both 95% and 99% CIs.

**Table 1.** Permutation test for baseline (pre-event) differences in nucleus accumbens dopamine release between groups at 95% and 99% confidence intervals

Groups	95% CI-baseline (pre-event) *p* value			
	No threshold	Consecutive threshold (2)	Consecutive threshold (4)	Consecutive threshold (5)
NonFoodObject versus Chow	0.952	0.948	0.951	0.957
NonFoodObject versus PeanutButter	0.059	0.061	0.058	0.068
Chow versus PeanutButter	0.027	0.027	0.031	0.03
	99% CI-baseline (pre-event) *p* value			
	No threshold	Consecutive threshold (2)	Consecutive threshold (4)	Consecutive threshold (5)
NonFoodObject versus Chow	0.945	0.955	0.974	0.95
NonFoodObject versus PeanutButter	0.065	0.053	0.075	0.064
Chow versus PeanutButter	0.041	0.027	0.027	0.032

For post-event differences between groups, *p* values only change slightly as the consecutive threshold increases (Table 2). The analysis calculates *p* values up to three decimal points; hence a *p* value of 0 simply means that it is <0.001. In general, these results highlight that the various thresholding approaches have little to no effect of permutation test *p* values. This is because permutation test considers all data points in the baseline or post-event period, where the consecutive threshold approach is not relevant. The slight variation in *p* values obtained from the different thresholds is due to the random sampling of data each time. Such that it is unlikely to obtain the same *p* values each time you run the permutation tests.

**Table 2.** Permutation test for pre- and post-event differences in nucleus accumbens dopamine release between groups using 95% and 99% confidence intervals

Groups	95% CI-comparison (post-event) *p* value			
	No threshold	Consecutive threshold (2)	Consecutive threshold (4)	Consecutive threshold (5)
NonFoodObject versus Chow	0.022	0.029	0.032	0.027
NonFoodObject versus PeanutButter	0	0	0	0.001
Chow versus PeanutButter	0.119	0.119	0.104	0.104
	99% CI-comparison (post-event) *p* value			
	No threshold	Consecutive threshold (2)	Consecutive threshold (4)	Consecutive threshold (5)
NonFoodObject versus Chow	0.027	0.022	0.026	0.016
NonFoodObject versus PeanutButter	0	0.001	0.001	0.001
Chow versus PeanutButter	0.104	0.094	0.106	0.103

## Discussion

Currently, statistical analysis of in vivo fiber photometry data is unintentionally flawed because it often uses biased user-defined summary statistics for peri-event time bins, such as area under the curve, average *z*-score, or peak *z*-score. Therefore, analytical tools to improve statistical reliability in an unbiased manner are essential for data reproducibility. We propose that a standardized easy-to-use post hoc analysis tool with less statistical assumptions would benefit the field. Indeed, with current approaches, changing time bin periods for area under curve analysis of average *z*-score may change the significance of the results and therefore the conclusions drawn from the experiments. This inadvertently biased approach hinders the field for multiple reasons including different researchers selecting different time bin periods or different parameters to indicate group differences, such as area under the curve, average *z*-score, and max peak *z*-score. Our bootstrapping CI, based on Jean-Richard-dit-Bressel and colleagues’ approach, removes the need to select the individual parameters or time bin period and thus, provides an unbiased analysis to strengthen the reliability of research outcomes (Jean-Richard-dit-Bressel et al., 2020). Recently, another post hoc analysis tool was published (Loewinger et al., 2025); however, this approach relies on data modeling with important assumptions about data distribution, such as convolving to a linear distribution, which may not be appropriate for the noisy, nonlinear, and nonparametric nature of fiber photometry signals. This approach is likely to be computationally demanding, which may hinder its use for large datasets. Regression-based approaches have been used previously and can be helpful for modeling fiber photometry data especially when used to study learning across smaller timescales (Mohebi et al., 2019; Lak et al., 2020). Given this approach, it may be useful in conjunction with FiPhoPHA for specific research questions as previously published (Hart et al., 2024). However, perhaps the main advantage of our approach is the usability and ease of adoption that we have intentionally built into the analysis. Our approach does not require a strong coding background and can be simply applied by installing the fiphopha package in Python. The graphical user interface enables the user to logically choose and execute the relevant analyses, and the data summary sheets expedite the presentation of statistical differences.

Our reanalysis of data taken from our previous study (Reichenbach et al., 2022) using the bootstrapping confidence intervals highlights greater statistical significance with greater temporal precision than 20 s time bin windows previously analyzed. For example, dopamine release to peanut butter is significantly different from the nonfood object in both time-binned analysis and bootstrapping. However, our new bootstrapping confidence interval analysis highlights significant differences between the nonfood object and chow, as well as between chow and peanut butter, which were not observed with our previous time-binned analysis. The time bin approach loses the ability to detect significance at precise time periods because time bins condense hundreds of data points into one data point. Our 20 s time bins from Reichenbach et al. (2022) represent condensing 200 data points sampled at 10 Hz into one data point (0.05 Hz), and therefore it is not surprising that our bootstrapping confidence interval analysis has identified additional significance at precise time periods between additional groups. Thus, our new analysis approach has identified that although the conclusions from Reichenbach et al. (2022) are valid, they are not as accurate as they could have been and left important statistical information undiscovered.

For our analysis pipeline, we recommend taking either the entire period before event onset or the entire recording period as the baseline value. Since the bootstrapped CIs have the possibility of type 1 errors, we recommend using the most conservative consecutive threshold measure out of the low-pass filter applied during data collection or the kinetics of the sensor. We have also incorporated analysis for between- and within-subjects intergroup comparisons that should be chosen accordingly. Depending on your specific physiological application, it may be more appropriate to use a standardized threshold or 99% CI versus 95% CI. Since the 99% CI uses a larger range of confidence of the signal at each individual time point, it allows for a more accurate measurement in some cases. In conjunction, the permutation test *p* values provide a statistical interrogation of the distribution of data during each period. Furthermore, it is important to not extrapolate beyond the given data and to recognize that the permutation tests for the baseline and comparison period give an exact *p* value, but the number of permutations that is chosen will change the smallest possible *p* value. Thus, we suggest that researchers consider increasing the number of permutations when dealing with a smaller sample size. Regardless, coupled with the bootstrapped CIs, this provides measurements of significance from multiple tests.

All analyses were performed on a 13th generation 14 core Intel Core i9-13900H CPU with 16 GB of RAM. Most of the tests are not computationally demanding except for the bootstrapped CIs and permutation tests, and the extent of the demand is dependent on the amount of data. Multiprocessing is incorporated in the code to utilize all cores of a computer's processing power; for this reason, we recommend running the code with limited background applications/processes concurrently running. The bootstrapped CIs and permutation tests took ∼1–2 min with the previously described computer specifications, during which, 60–100% of the CPU was used and 70% of RAM. For this case we recommend having a computer with at least 8 GB of RAM. If larger datasets become too computationally demanding, we recommend downsampling the data to a greater extent or reducing the amount of resamples and permutations.

### Conclusion

The various packages available for fiber photometry analysis lack usability, accessibility, and objective post hoc statistical methods. Here we introduce FiPhoPHA as a Python package that performs post hoc analyses on any raw dataset. FiPhoPHA serves as post hoc analysis toolkit to improve reliability and data reproducibility in an unbiased manner for all photometry users. This package incorporates a downsampler, mean time bins, bootstrapped Cis, and permutation tests.

## Synthesis

Reviewing Editor: Mark Laubach, American University Decisions are customarily a result of the Reviewing Editor and the peer reviewers coming together and discussing their recommendations until a consensus is reached. When revisions are invited, a fact-based synthesis statement explaining their decision and outlining what is needed to prepare a revision will be listed below. The following reviewer(s) agreed to reveal their identity: Talia Lerner. Note: If this manuscript was transferred from JNeurosci and a decision was made to accept the manuscript without peer review, a brief statement to this effect will instead be what is listed below.

Your revisions were reviewed by the same people. One reviewer signed off and thought that you addressed all concerns. The other raised two points that you should address and that will be handled by editorial review. The reviewer's full review is given below.

Please address two points:

1. Please answer the reviewer's questions in point 1 How does your method deal with unbalanced data across types of trials or trials by subject?

2. Please revise the papers cited in the introduction to the paper, as suggested by the reviewer.

Reviewer comments:

1. In the input Excel file, each column could be subjects or trials. If trials, then the analysis pulls all subjects together? How does having subjects or trials as columns affect the analysis? In particular, how does that affect the bootstrapped confidence intervals and permutation tests?

Author's rebuttal:

This is a common misconception based on traditional photometry analysis, whereby researchers can sometimes report large sample sizes from presenting and analyzing multiple trials from the same animal. This analysis circumnavigates this problem by allowing smaller sample sizes to be run and sampled thousands of times. For instance, if researchers only had 5 animals in 2 different groups, they would be able to draw statistically valid conclusions on a time by timepoint scale due to the statistical power of bootstrapping. Also, we have also incorporated an adjustment for narrowness bias as mentioned in the introduction, in addition researchers can increase resamples and permutations with smaller sample sizes to increase accuracy.

Reviewer 2 follow-up:

Thank you for the answer. However, in some experimental setups, like during decision-making tasks, you often get an unequal number of trials and/or trial types per subject. As far as I understand, bootstrapping at the trial-level, the subject with a higher number of trials will dominate, which will result in overly tight confidence intervals and overly small p-values.

Also, to the point that researchers can increase resamples and permutations with smaller sizes to increase accuracy, that is, still with the caveat that data should be homogeneous. If the data is very variable, you won't automatically get smaller confidence intervals. I believe it would be useful for the reader to add these caveats, or points to take into consideration when running this analysis.

2. Comparison with previous analysis: The authors claim that statistical analysis of photometry data is flawed because of user-defined statistics and because of the use of assumptions about the data that might not be appropriate. Can the authors provide specific examples or expand on this?

Authors' rebuttal:

We have now included some specific examples of user-defined statistical analysis in the introduction.

Indeed, almost every paper with photometry data, including our own, have used time binned averages, average max Z-score or area under the curve analysis because no other alternative was available. These are all inherently, but unintentionally biased, based on the need to choose a time frame associated with the analysis. The bootstrapped confidence intervals and permutation tests function independent from any temporal information within the dataset and is the key reason why we have developed this easy to use python package.

Reviewer 2 follow-up:

All the 'flawed' examples the authors now cite in their introduction come from the authors' lab. First, I would suggest including citations from other groups. Second, it isn't true that almost every photometry paper only uses binned averages or AUC because no alternative was available. There are many examples of many different groups that they also use other methods, like timepoint-by-timepoint regression models. For example, see Parker et al., 2016 (https://doi.org/10.1038/nn.4287), Lee et al., 2019 (https://doi.org/10.7554/eLife.42992), Mohebi et al., 2019 (https://doi.org/10.1038/s41586-019-1235-y), Lak et al., 2020 (https://doi.org/10.1016/j.neuron.2019.11.018), Kim et al., 2020 (https://doi.org/10.1016/j.cell.2020.11.013), Hart et al., 2024 (https://doi.org/10.1016/j.celrep.2024.113828), or Simpson et al., 2024 (https://doi.org/10.1016/j.neuron.2023.11.016), for a review of these other methods for post-processing photometry data, which the authors cite in their introduction.

Related to these regression methods to analyze photometry data, the authors' main critique of Loewinger et al., 2025 tool is the complexity and the assumptions which they claim are not appropriate for photometry, but 1) every method has assumptions, and there are different diagnostics that can be checked on the mixed-effects models to check the appropriateness of the method, plus there are different ways to handle non-linearities. 2) regression-based approaches can handle covariates and unbalanced data, that's why many of the above studies use them. And 3) Loewinger et al., 2025 directly compared their joint CIs from mixed-effects against bootstrap CIs (as in Jean-Richard-dit-Bressel et al., 2020, and consequently, in FiPhoPHA), and found joint CIs to be a better metric. Therefore, I don't agree with the authors that Loewinger et al., 2025 tool is inappropriate to analyze photometry data.

That being said, I agree FiPhoPHA is a valuable, user-friendly tool, and can even complement these more complex analyses. For example, Hart et al., 2024 (https://doi.org/10.1016/j.celrep.2024.113828) used Jean-Richard-dit-Bressel et al., 2020 bootstrapped confidence intervals procedure on both mean signals and regression coefficients, highlighting that bootstrapped CIs can be a good statistical approach for photometry data, but this is independent from whether a mixed effect or fixed effect regression analysis is being performed.

## Author Response

Synthesis of Reviews:

Please address two points:

1. Please answer the reviewer's questions in point 1 How does your method deal with unbalanced data across types of trials or trials by subject? 2. Please revise the papers cited in the introduction to the paper, as suggested by the reviewer.

Reviewer comments:

1. In the input Excel file, each column could be subjects or trials. If trials, then the analysis pulls all subjects together? How does having subjects or trials as columns affect the analysis? In particular, how does that affect the bootstrapped confidence intervals and permutation tests? Author's rebuttal:

This is a common misconception based on traditional photometry analysis, whereby researchers can sometimes report large sample sizes from presenting and analyzing multiple trials from the same animal. This analysis circumnavigates this problem by allowing smaller sample sizes to be run and sampled thousands of times. For instance, if researchers only had 5 animals in 2 different groups, they would be able to draw statistically valid conclusions on a time by timepoint scale due to the statistical power of bootstrapping. Also, we have also incorporated an adjustment for narrowness bias as mentioned in the introduction, in addition researchers can increase resamples and permutations with smaller sample sizes to increase accuracy.

Reviewer 2 follow-up:

Thank you for the answer. However, in some experimental setups, like during decision-making tasks, you often get an unequal number of trials and/or trial types per subject. As far as I understand, bootstrapping at the trial-level, the subject with a higher number of trials will dominate, which will result in overly tight confidence intervals and overly small p-values.

Our response This is an excellent point and for this case bootstrapping at the level of the subject would be more appropriate. The choice of analysing by either trial or subject is important (Recommendations for the Design and Analysis of in vivo Electrophysiology Studies (2018). J. Neurosci. 38, 5837-5839. 10.1523/jneurosci.1480-18.2018). Using trial data as you state can introduce some bias due to unbalanced trial counts across subjects, and inflate certain statistical outcomes, but in some cases it is also more appropriate, especially if data is balanced and depending on research question. Hence it is important for researchers to determine what type of data to input into the package based on research question. We have added this consideration into the methods. "The choice of analysing by animal or trial depends on the researcher's specific question. We note that unbalanced trial data can inflate the reliability of an effect but may be appropriate in certain circumstances (Editorial, 2018). Hence it is important for researchers using this package to choose between or within-subjects comparisons using animal/trial data according to their specific data and the analysis required".

Also, to the point that researchers can increase resamples and permutations with smaller sizes to increase accuracy, that is, still with the caveat that data should be homogeneous. If the data is very variable, you won't automatically get smaller confidence intervals. I believe it would be useful for the reader to add these caveats, or points to take into consideration when running this analysis.

Our response We appreciate the reviewers analysis and comments on bootstrap and permutation size. Increasing the number of bootstraps or permutations does not narrow the CI. Rather it provides a more representative bootstrapped distribution, which asymptotes quickly, hence 2000 bootstraps may not necessarily be better than 1000 (Jean-Richard-Dit-Bressel P, Clifford CWG, McNally GP. Analyzing Event-Related Transients: Confidence Intervals, Permutation Tests, and Consecutive Thresholds. Front Mol Neurosci. 2020 Feb 6;13:14. doi: 10.3389/fnmol.2020.00014.). If the data is unbalanced and of a low sample size, the distribution will be wider but as the sample size increases the CI will become narrower, even whilst unbalanced as it becomes closer to approximate the true population. Hence the variability and heterogeneity of the data is not a caveat, and bootstrapping is actually is the most appropriate test to provide a closer population estimate.

We have added the following to the methods.

It should be noted that increasing the number of bootstraps or permutations does not narrow the CI. Rather it provides a more representative bootstrapped distribution, which asymptotes quickly, hence 2000 bootstraps may not necessarily be better than 1000.

2. Comparison with previous analysis: The authors claim that statistical analysis of photometry data is flawed because of user-defined statistics and because of the use of assumptions about the data that might not be appropriate. Can the authors provide specific examples or expand on this? Authors' rebuttal:

We have now included some specific examples of user-defined statistical analysis in the introduction.

Indeed, almost every paper with photometry data, including our own, have used time binned averages, average max Z-score or area under the curve analysis because no other alternative was available. These are all inherently, but unintentionally biased, based on the need to choose a time frame associated with the analysis. The bootstrapped confidence intervals and permutation tests function independent from any temporal information within the dataset and is the key reason why we have developed this easy to use python package.

Reviewer 2 follow-up:

All the 'flawed' examples the authors now cite in their introduction come from the authors' lab. First, I would suggest including citations from other groups. Second, it isn't true that almost every photometry paper only uses binned averages or AUC because no alternative was available. There are many examples of many different groups that they also use other methods, like timepoint-by-timepoint regression models. For example, see Parker et al., 2016 (https://doi.org/10.1038/nn.4287), Lee et al., 2019 (https://doi.org/10.7554/eLife.42992), Mohebi et al., 2019 (https://doi.org/10.1038/s41586-019-1235-y), Lak et al., 2020 (https://doi.org/10.1016/j.neuron.2019.11.018), Kim et al., 2020 (https://doi.org/10.1016/j.cell.2020.11.013), Hart et al., 2024 (https://doi.org/10.1016/j.celrep.2024.113828), or Simpson et al., 2024 (https://doi.org/10.1016/j.neuron.2023.11.016), for a review of these other methods for post-processing photometry data, which the authors cite in their introduction.

Our response These are excellent points, and we have now added citations from other groups as well. We have also cited some articles that use regression based analyses in the discussion.

Regression based approaches have been used previously and can be helpful for modelling fiber photometry data especially when used to study learning across smaller timescales (Mohebi et al., 2019; Lak et al., 2020). Given this approach, it may be useful in conjunction with FiPhoPHA for specific research questions as previously published (Hart et al., 2024).

Related to these regression methods to analyze photometry data, the authors' main critique of Loewinger et al., 2025 tool is the complexity and the assumptions which they claim are not appropriate for photometry, but 1) every method has assumptions, and there are different diagnostics that can be checked on the mixed-effects models to check the appropriateness of the method, plus there are different ways to handle non-linearities. 2) regression-based approaches can handle covariates and unbalanced data, that's why many of the above studies use them. And 3) Loewinger et al., 2025 directly compared their joint CIs from mixed-effects against bootstrap CIs (as in Jean-Richard-dit-Bressel et al., 2020, and consequently, in FiPhoPHA), and found joint CIs to be a better metric. Therefore, I don't agree with the authors that Loewinger et al., 2025 tool is inappropriate to analyze photometry data.

Our response We thank the reviewer for pointing out the strengths of regression-based approaches and general suitability for fiber photometry especially by Loewinger et al., 2025. Hence we have added some points to the discussion that this approach is useful, especially in conjunction with our approach combined.

Regression based approaches have been used previously and can be helpful for modelling fiber photometry data especially when used to study learning across smaller timescales (Mohebi et al., 2019; Lak et al., 2020).

That being said, I agree FiPhoPHA is a valuable, user-friendly tool, and can even complement these more complex analyses. For example, Hart et al., 2024 (https://doi.org/10.1016/j.celrep.2024.113828) used Jean-Richard-dit-Bressel et al., 2020 bootstrapped confidence intervals procedure on both mean signals and regression coefficients, highlighting that bootstrapped CIs can be a good statistical approach for photometry data, but this is independent from whether a mixed effect or fixed effect regression analysis is being performed.

Our response Thank you for this useful constructive feedback, which helps to clarify our method and paper. We have added the potential to use regression analysis with FiPhoPHA in the discussion as pointed out by the reviewer.

Regression based approaches have been used previously and can be helpful for modelling fiber photometry data especially when used to study learning across smaller timescales (Mohebi et al., 2019; Lak et al., 2020). Given this approach, it may be useful in conjunction with FiPhoPHA for specific research questions as previously published (Hart et al., 2024).

Editorial (2018) Recommendations for the Design and Analysis of In Vivo Electrophysiology Studies. The Journal of neuroscience 38:5837-5839.

Hart G, Burton TJ, Nolan CR, Balleine BW (2024) Striatal dopamine release tracks the relationship between actions and their consequences Striatal dopamine release tracks the relationship between actions and their consequences. Cell reports (Cambridge) 43:113828.

Lak A, Okun M, Moss MM, Gurnani H, Farrell K, Wells MJ, Reddy CB, Kepecs A, Harris KD, Carandini M (2020) Dopaminergic and Prefrontal Basis of Learning from Sensory Confidence and Reward Value. Neuron (Cambridge, Mass) 105:700-711.e706.

Mohebi A, Pettibone JR, Hamid AA, Wong J-MT, Vinson LT, Patriarchi T, Tian L, Kennedy RT, Berke JD (2019) Dissociable dopamine dynamics for learning and motivation. Nature (London) 570:65-70.
